# Combining alpha radiation-based brachytherapy with immunomodulators promotes complete tumor regression in mice via tumor-specific long-term immune response

**DOI:** 10.1007/s00262-019-02418-5

**Published:** 2019-10-22

**Authors:** Vered Domankevich, Adi Cohen, Margalit Efrati, Michael Schmidt, Hans-Georg Rammensee, Sujit S. Nair, Ashutosh Tewari, Itzhak Kelson, Yona Keisari

**Affiliations:** 1 Department of Clinical Microbiology and Immunology, Sackler Faculty of Medicine, Tel Aviv University, P.O. Box 39040, 6997801 Tel Aviv, Israel; 2 School of Physics and Astronomy, Sackler Faculty of Exact Sciences, Tel Aviv University, Tel Aviv, Israel; 3 Alpha Tau Medical, Tel Aviv, Israel; 4 Department of Immunology, Institute for Cell Biology, University of Tübingen, Tübingen, Germany; 5 Department of Urology, Icahn School of Medicine at Mount Sinai, New York, NY USA

**Keywords:** Tumor ablation, Poly I:C, CpG, XS15, Treg, MDSC

## Abstract

Diffusing alpha-emitters radiation therapy (DaRT) is the only known method for treating solid tumors with highly destructive alpha radiation. More importantly, as a monotherapy, DaRT has been shown to induce a systemic antitumor immune response following tumor ablation. Here, immunomodulatory strategies to boost the antitumor immune response induced by DaRT, and the response specificity, were investigated in the colon cancer CT26 mouse model. Local treatment prior to DaRT, with the TLR3 agonist poly I:C, was sufficient to inhibit tumor growth relative to poly I:C or DaRT alone. DaRT used in combination with the TLR9 agonist CpG, or with the TLR1/2 agonist XS15 retarded tumor growth and increased tumor-rejection rates, compared to DaRT alone, curing 41% and 20% of the mice, respectively. DaRT in combination with CpG, the Treg inhibitor cyclophosphamide, and the MDSC inhibitor sildenafil, cured 51% of the animals, compared to only 6% and 0% cure when immunomodulation or DaRT was used alone, respectively. Challenge and Winn assays revealed that these high cure rates involved a specific immunological memory against CT26 antigens. We suggest that DaRT acts in synergy with immunomodulation to induce a specific and systemic antitumor immune response. This strategy may serve as a safe and efficient method not only for tumor ablation, but also for in situ vaccination of cancer patients.

## Introduction

Ablation strategies are non-surgical procedures that destroy solid tumors in situ, thereby releasing tumor antigens and damage-associated molecular pattern (DAMP) molecules, which then promote a systemic antitumor immune response (for review, see [1]). A unique method for solid tumor ablation using highly destructive alpha radiation was developed in our laboratories, termed diffusing alpha-emitters radiation therapy (DaRT) [2, 3]. The treatment was found to destroy mouse and human tumors of squamous cell carcinoma [4, 5], lung carcinoma [6], prostate, glioblastoma, colon [7], and pancreatic [8] tumors. By widening the range of the alpha-emitting atoms’ distribution inside the tumor, this method provides the only known application for alpha-based brachytherapy of solid tumors.

Synergy between radiotherapy and immunotherapy has been demonstrated in preclinical trials, and a mechanistic rationale to combine both therapies has been suggested. Radiation increases antigen visibility, enhances MHC1 expression, and promotes phagocytosis by antigen-presenting cells, thereby leading to T-cell priming, antigen-specific recognition and, in some cases an abscopal effect [9]. Specifically, particle radiation, such as proton and carbon ions, induces an antitumor response, resulting in the suppression of distant metastases [10]. However, immune activation by radiation is strictly regulated to prevent unwanted recognition of self-antigens, for example, by recruitment of MDSCs to the radiated site [9]. Thus, methods to boost antitumor immunity induced by radiation include strategies to counteract the immunosuppressive tumor microenvironment. Another possibility is to stimulate the immune response induced by radiotherapy using immunoadjuvants that may activate dendritic cells and promote antigen cross-priming [11].

Tumor ablation by DaRT has been shown to induce a systemic antitumor immune response against tumor cells. In the colorectal carcinoma CT26 mouse model, DaRT protected against tumor challenge in both the skin and lungs, and in the murine mammary cancer model, DA3, DaRT protected against skin tumor challenge and inhibited lung metastases [12]. Furthermore, DaRT-mediated systemic antitumor immune response is significantly enhanced when combined with immunomodulators. In the DA3 breast cancer model, DaRT combined with the TLR9 agonist CpG further retarded tumor growth compared to each treatment alone [12]. Combining DaRT with Treg/MDSC inhibitors protected against primary/challenge tumor development [13], whereas DaRT combined with CpG and Treg/MDSC inhibitors reduced lung metastases and enhanced the tumor response, leading to complete tumor rejection.

In the present study, we used the colon CT26 tumor model to investigate two main scientific questions: (1) Can other types of TLR agonists be used in combination with DaRT to stimulate antitumor immunity? [2] What is the nature and specificity of the immune response triggered by DaRT when combined with immunostimulation and inhibition of suppressor immune cells?

## Materials and methods

### Animals

BALB/c female mice (~ 20 g, 10 weeks old) were kept in the animal facility at Tel Aviv University. All surgical and invasive procedures were performed under anesthesia using ketamine (100 mg/kg, Bremer Pharma, Germany) and xylazine hydrochloride (10 mg/kg, Eurovet Animal Health B.V., Bladel, Netherlands) solution in phosphate-buffered saline (PBS). An i.p. injection was given 10 min before starting the treatment.

### Tumor cell lines

All cell lines were stored in a humid incubator at a temperature of 37 °C and 5% CO_2_. CT26 cells were grown in RPMI-1640 containing l-glutamine, supplemented with 10% fetal calf serum, penicillin (100 U/ml), streptomycin (100 μg/ml), nystatin (12.5 U/ml), sodium pyruvate (1 mM), and HEPES buffer (1 M) (Biological Industries, Kibbutz Beit Haemek, Israel).

DA3 cells were grown in Dulbecco’s modified Eagle’s medium containing 4.5 g/l d-glucose and 4 mM l-glutamine, supplemented with 10% fetal calf serum, penicillin (100 U/ml), streptomycin (100 μg/ml), and nystatin (12.5 U/ml) (Biological Industries).

### Tumor cell inoculation

Mice were inoculated i.d. with 5 × 10^5^ cells, unless otherwise specified, into the low lateral side of the back in 0.05 ml RPMI or Hanks’ balanced salt solution (HBSS) (Biological Industries).

### Tumor volume measurements

Local tumor growth was determined by measuring three mutually orthogonal tumor dimensions two to three times per week, according to the following formula: Tumor volume = *π*/6 × diameter 1 × diameter 2 × height. For cumulative data of two or more experiments, extrapolation using the TREND function in Excel (based on the two closest existing measurements) was used in the case of missing corresponding measurement time points.

### Immunomodulators preparation

CpG (Syntezza, Jerusalem, Israel) was dissolved in PBS to the indicated concentrations. PBS served as the control. Cyclophosphamide (CP) (Sigma, Israel) was dissolved in saline. Saline served as the control. Sildenafil (Pfizer, NY) was prepared as previously described [14]. Glass bottles were used to avoid absorption and bottles were covered with aluminum foil to protect from the light. Bottles were shaken five times a week, and the solution was exchanged twice a week. XS15 (N-palmitoyl-S-[2,3-bis(palmitoyloxy)-(2R)-propyl]-(R)-cysteinyl-GDPKHPKSF) (EMC Microcollections, Tübingen, Germany) [15] was dissolved in sterile endotoxin-free water according to the manufacturer’s instructions. High-molecular-weight poly I:C (InvivoGen, Toulouse, France) was dissolved in PBS or physiological water according to the manufacturer’s instructions. All of the reagents for injection were prepared using sterile solutes in a biohazard hood.

### Winn assay

Spleens were harvested, immersed in PBS, ground with the flat end of a syringe and passed through a cell strainer. Cells were washed in RPMI/HBSS and centrifuged at 394*g* for 7 min. The supernatant was removed, and cells were resuspended and pooled. Red blood cells were lysed, and cells were washed in HBSS. Cells were then mixed with tumor cells in the indicated concentrations and immediately injected in a volume of 0.2 ml.

### ^224^Radium (Ra)-loaded seed (DaRT seed) preparation and insertion

Stainless steel wires (0.4 mm diameter, 6–8 mm length) were loaded with ^224^Ra atoms (half-life of 3.7 days). To prevent Ra dissolution in the tissue fluids, the atoms were embedded a few atomic layers into the seed surface through thermal treatment [2]. Seeds, either loaded with ^224^ Ra or inert, were placed near the tip of a 21-gauge needle attached to an insertion applicator. The radioactive and inert seeds were inserted into the primary tumor under anesthesia.

### Statistical analysis

Statistical significance (*p* value) was determined by two-tailed Student’s *t* test for comparisons of group means or by χ^2^ test for comparisons of proportions between experimental groups.

## Results

### Combined treatment with DaRT and the TLR3 agonist poly I:C retards CT26 tumor development compared to each treatment alone

**Fig. 1:**
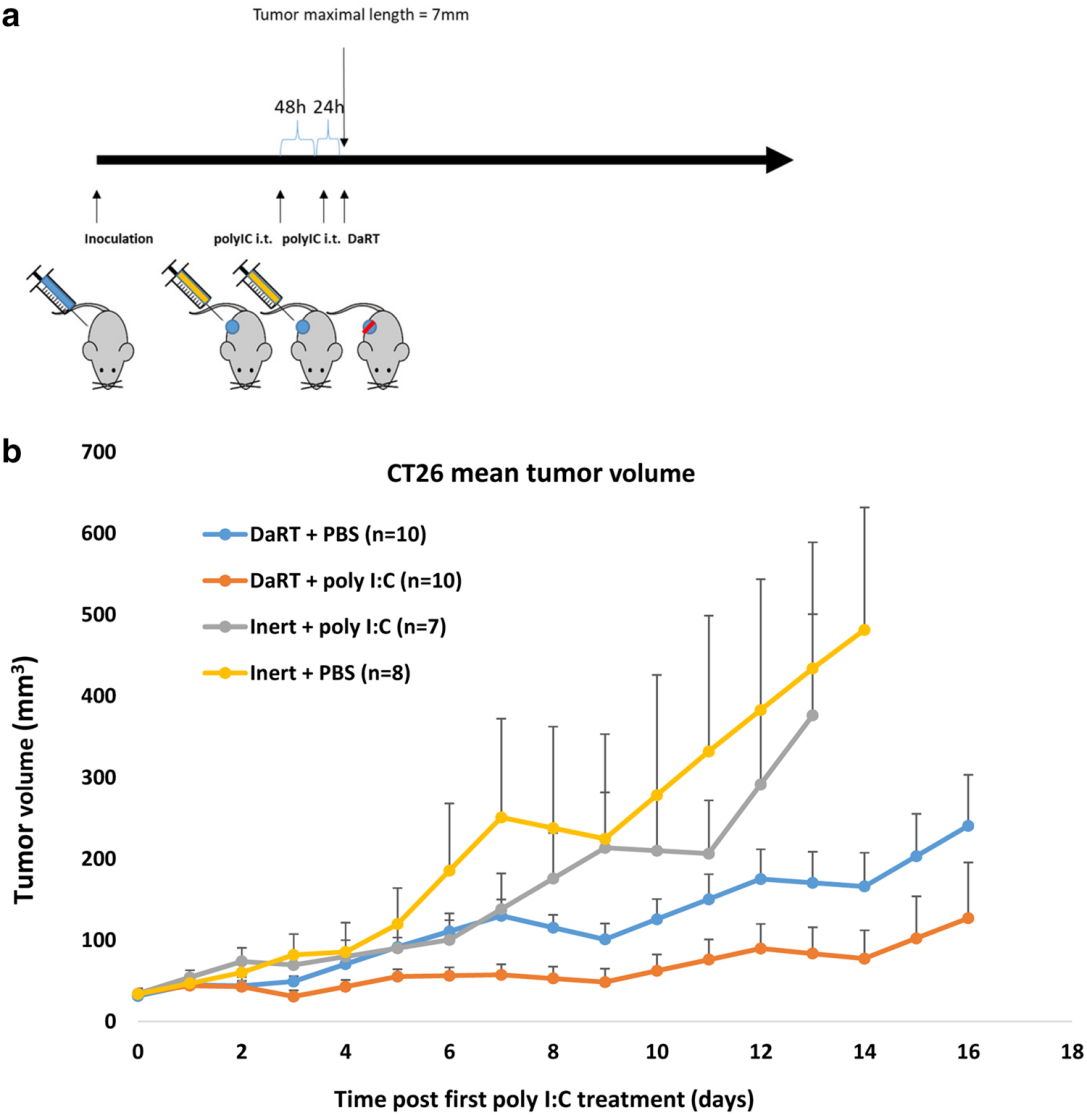
CT26 tumor development following DaRT + TLR3 agonist. **a** Experimental scheme. CT26-bearing mice were treated with either a 40 kBq DaRT seed or inert seed; 72 and 24 h prior to seed insertion, an intratumoral (i.t.) injection of 10 µg/30 µl poly I:C or PBS was given. **b** Tumor growth curve. DaRT + poly I:C reduced tumor growth compared to all other groups. *p*_*t* test_ < 0.05 for DaRT + poly I:C vs. control on days 3, 13 and 14, vs. DaRT alone on days 4–8, and vs. poly I:C alone on days 3, 8–10, 12, 13

The effect of DaRT combined with the TLR3 agonist poly I:C on tumor development was investigated in the immunogenic CT26 tumor model. The poly I:C (10 µg/30 µl) was injected intratumorally into CT26-bearing mice 72 h and 24 h prior to DaRT treatment. When tumor maximal length reached 7.4 mm, a 7-mm 40 kBq DaRT seed was inserted into the tumor (Fig. 1a). DaRT and local poly I:C treatments were sufficient to significantly retard tumor growth compared to all other groups (Fig. 1b) (*p*_*t* test_ < 0.05 for DaRT + poly I:C vs. control on days 3, 13, 14, DaRT alone on days 4–8, and poly I:C alone on days 3, 8–10, 12, 13). Notably, DaRT alone significantly retarded tumor growth (*p*_*t* test_ < 0.05) on days 13 and 14, whereas poly I:C alone was not significantly different from the control at any of the time points.

### Combined treatment with DaRT and the TLR9 agonist CpG, or the TLR1,2 agonist XS15, retards CT26 tumor development and increases tumor-rejection rates compared to DaRT alone

**Fig. 2:**
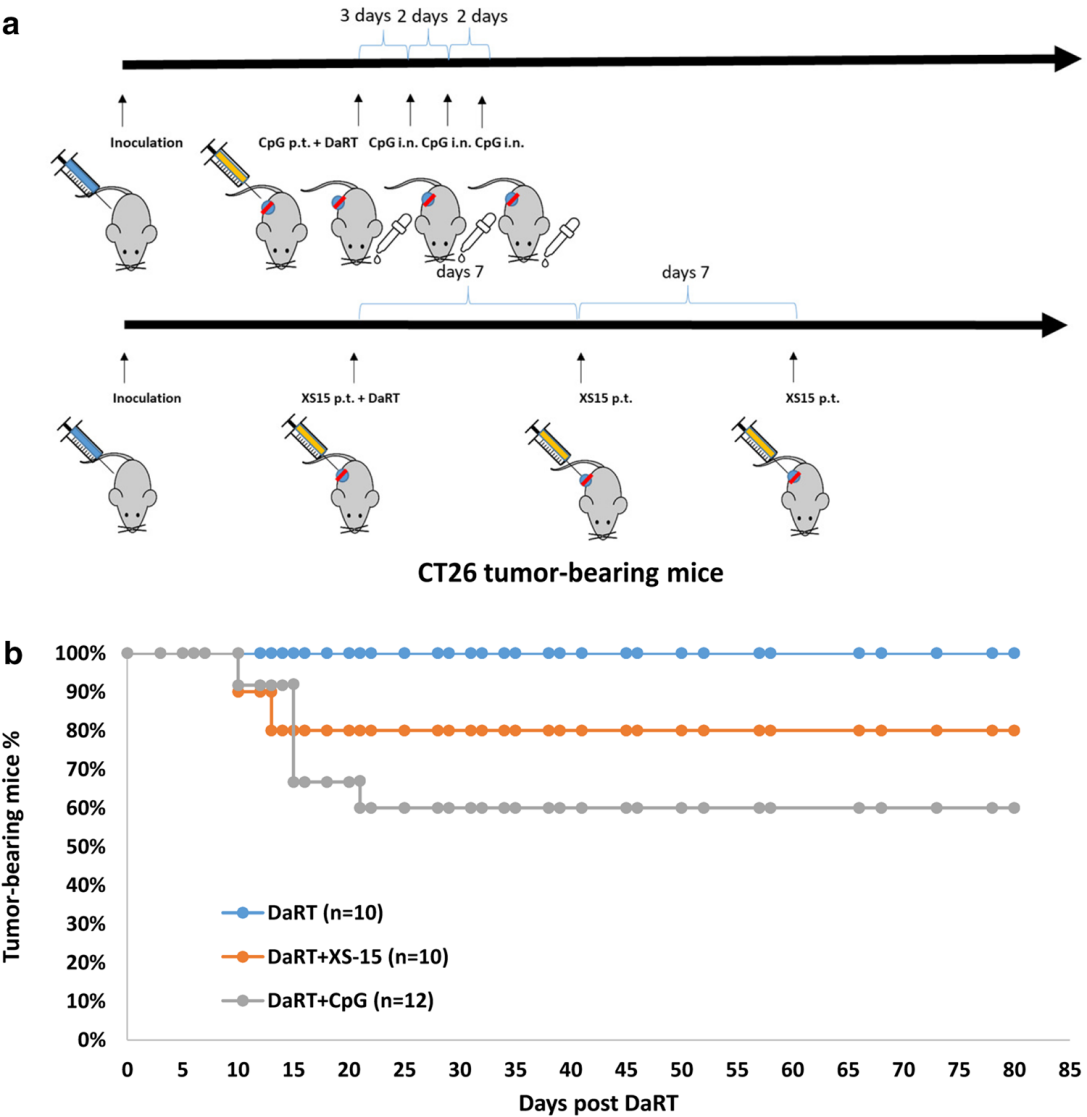
CT26 tumor rejection following DaRT combined with TLR9/TLR1,2 agonists. **a** Experimental scheme. CT26-bearing mice were treated with a 40–50 kBq DaRT seed in combination with CpG or XS15. For XS15 treatment, a 40 µg/50 µl peritumoral (p.t.) injection was given once a week for 3 weeks (three injections, first treatment on the day of DaRT insertion). For CpG treatment, a 100 µg/30 µl p.t. injection was given immediately prior to DaRT insertion and 20 µg/10 µl was administered intranasally (i.n.) three times thereafter, every 2 days. **b** Percent of tumor-bearing mice. *p*_χ2 test_ < 0.05 for DaRT vs. DaRT + CpG. The presented results are based on cumulative data from two different experiments

The above results agreed with our previous findings in the DA3 breast cancer model showing that DaRT in combination with the TLR9 agonist CpG significantly inhibits tumor development compared to each of the treatments alone [12]. We also confirmed the enhancement of DaRT-induced tumor retardation by CpG in the CT26 tumor model. In addition, potential enhancement by the TLR1,2 agonist XS15 was examined. CT26-bearing mice were treated with a 100 µg/30 µl peritumoral injection of CpG immediately prior to DaRT insertion, and then a dose of 20 µg/10 µl CpG administered intranasally three times (once every 2 days), starting 3 days after DaRT insertion, or with a 40 µg/50 µl peritumoral injection of XS15 once a week for 3 weeks from the day of DaRT insertion (Fig. 2a). A 7-mm-long DaRT seed (40–50 kBq) was inserted into the tumor when its maximal length reached 8–10 mm.

DaRT combined with either CpG or XS15 treatment retarded tumor development compared to DaRT alone. Tumor volume on day 10 for mice treated with DaRT was 738 ± 170 mm^3^; with DaRT + XS15: 252 ± 39 mm^3^; and for DaRT + CpG: 187 ± 61 mm^3^ (*p*_*t* test_ < 0.05 on days 6 and 10). In addition, DaRT combined with CpG or XS15 cured 5 out of 12 (41%) or 2 out of 10 (20%) of the mice, respectively. The cure rates for animals treated with DaRT combined with CpG were significantly different from those treated with DaRT alone (0% cure; *P*_χ2__test_ < 0.05; Fig. 2b).

### Combined treatment with DaRT, CpG, CP, and sildenafil inhibits CT26 tumor growth and increases cure rates compared to immunomodulation alone or DaRT alone

**Fig. 3:**
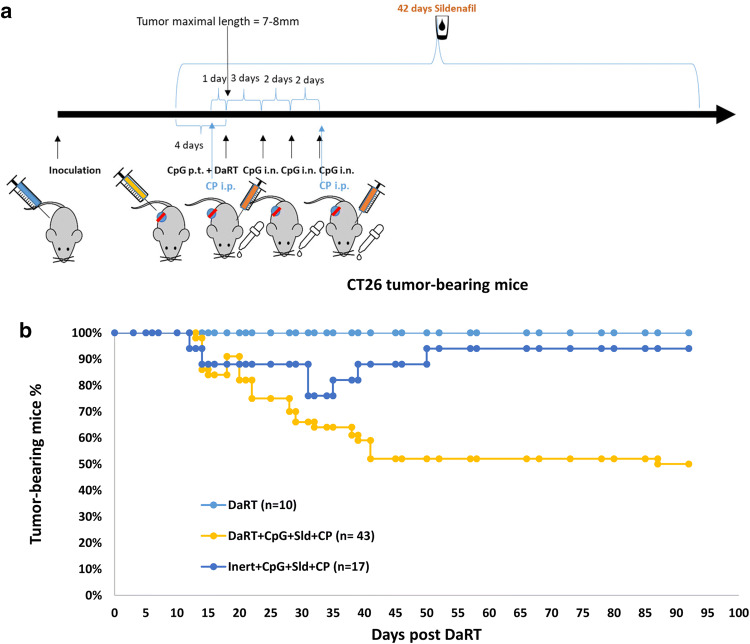
CT26 tumor rejection following DaRT combined with TLR9 agonist and MDSC/Treg inhibitors. **a** Schematic representation of the treatment protocol combining DaRT with immunomodulators. Mice were inoculated with 5 × 10^5^ CT26 cells. When tumor maximal length reached 7–8 mm (10–14 days after inoculation), a single 7-mm 60 kBq DaRT seed, or an inert seed, was inserted into the tumor. Four days prior to DaRT, systemic treatment with sildenafil was begun (0.33 mg/ml in the drinking water, daily for 6 weeks). An i.p. injection of 125 mg/kg CP was given 1 day before DaRT and 1 week after DaRT. On the day of DaRT treatment, 100 µg/30 µl CpG were administered peritumorally (p.t.) and three doses of 20 µg/10 µl CpG were given intranasally (i.n.) every 2 days starting from day 3 after DaRT. **b** Percent tumor-bearing mice during 3 months of follow-up. *p*_χ2 test_ < 0.005 for DaRT combined with the three immunomodulators vs. all other groups. The presented results are based on cumulative data from three different experiments

In a previous investigation of the DA3 breast cancer mouse model, we showed that inhibition of immunosuppressive cells by the Treg inhibitor CP and the MDSC inhibitor sildenafil further enhances DaRT-induced primary/challenge tumor retardation and inhibits the development of lung metastases when used with or without CpG [13]. We therefore studied DaRT in combination with the immunomodulators CpG, CP, and sildenafil in the CT26 tumor model. CT26-bearing mice were treated as follows: 4 days prior to DaRT treatment, a systemic treatment with sildenafil was begun (0.33 mg/ml in the drinking water, daily for 6 weeks), and an i.p. injection of 125 mg/kg CP was given 1 day prior to DaRT. On the day of the DaRT treatment, 100 µg/30 µl CpG were administered peritumorally and three doses of 20 µg/10 µl CpG were given intranasally every 2 days thereafter (Fig. 3a). At this time point, maximal tumor length was 7–8 mm. DaRT treatment was applied using a single 7-mm ^224^Ra seed (60 kBq). One week after DaRT insertion an additional dose of CP was given (Fig. 3a).

Tumor-rejection rates were significantly enhanced in animals treated with the three immunomodulators in combination with DaRT seeds, compared to the same treatment with inert seeds or DaRT alone (Fig. 3b). In the group treated with DaRT alone, there were no cured tumors. For mice treated with immunomodulators and an inert seed, 16 out of 17 (94% of the tumors) did not disappear or disappeared temporarily, whereas 22 out of 43 tumors (51%) were cured in the group treated with DaRT combined with immunomodulators up to 93 days following treatment. The difference was significant (*P*_χ2 test_ < 0.005) for DaRT combined with the three immunomodulators vs. all other groups (Fig. 3b). These results suggest that the immunomodulatory treatment and DaRT act synergistically to induce a long-term antitumor response.

In the group treated with DaRT and immunomodulators (Fig. 3b), 10% of the tumors that had been cured relapsed on day 15 post-DaRT—the last day of systemic CpG treatment. We therefore tested whether an extension of the systemic intranasal CpG treatment would improve tumor retardation. In this experiment, mice were treated using the same treatment regime as described above, yet in one treatment group the systemic CpG treatment was extended (6 doses given every 2 days instead of 3 doses every 2 days). CT26-bearing mice were treated with 60–70 kBq ^224^Ra DaRT seeds, combined with 100 µg/30 µl CpG injected peritumorally, three or six doses of 20 µg/10 µl CpG administered intranasally, 0.33 mg/ml sildenafil in the drinking water, and i.p. injection of 125 mg/kg CP. Inert seeds combined with immunomodulation that included the extended CpG treatment served as a control (*n* = 9). Corresponding with the results in Fig. 3, tumor-rejection rates in the group treated with DaRT and immunomodulators was 78% with the short CpG treatment, and 70% with the extended CpG treatment, vs. only 14% in the control group (inert seed + immunomodulators). Thus, extension of the systemic CpG treatment at this time point did not significantly improve primary tumor retardation. Primary tumor volume (mean ± SEM) on day 32 post-DaRT insertion was 90 ± 81 vs. 61 ± 42.5 mm^3^ in the short CpG group (*n* = 10) vs. the extended CpG group (*n* = 10), respectively (*p*_*t* test_ > 0.05).

### CT26-bearing mice cured by immunomodulation combined with DaRT, but not inert seed, show delayed tumor development when rechallenged with a higher number of cells

**Fig. 4:**
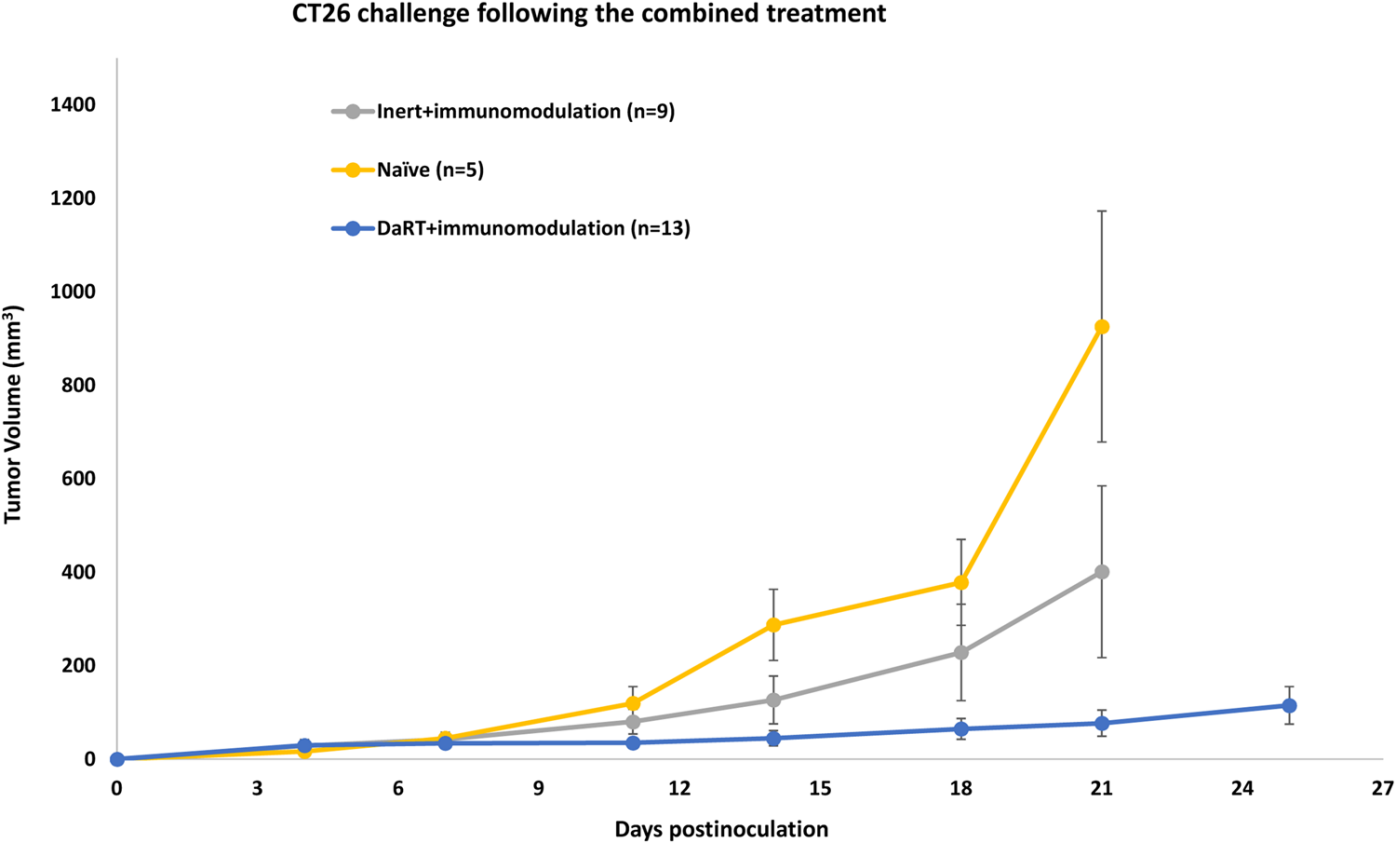
Rechallenge tumor development in CT26-bearing mice treated with DaRT vs. inert seed combined with immunomodulators. Mice were inoculated with 5 × 10^5^ CT26 tumor cells and then treated with DaRT or inert seeds combined with immunomodulators CpG, CP, and sildenafil. Cured mice or tumor-resected mice were rechallenged ~ 4 months after DaRT with 5 × 10^6^ CT26 tumor cells

The role of DaRT in inducing long-term antitumor immune memory when combined with immunomodulators (CpG, sildenafil, and CP) was then investigated. Mice were treated by immunomodulation in combination with either DaRT or inert seed. Cured mice or mice that underwent tumor resection when tumor volume exceeded 150 mm^3^ were rechallenged with a higher number of cells (5 × 10^6^ vs. 5 × 10^5^ used in the initial inoculation) ~ 4 months after DaRT insertion. An additional group of naïve mice served as controls. Mice that were treated with DaRT and the immunomodulators and then underwent tumor challenge, showed significantly retarded tumor growth compared to naïve mice inoculated with the same number of cells (5 × 10^6^; *p*_*t* test_ < 0.05, on days 14–21). In contrast, retardation of tumor growth in mice treated with immunomodulators and inert seeds, compared to naïve mice, was not significant. Challenged tumor volume on day 21 postinoculation for mice originally treated with DaRT and immunomodulators (*n* = 13), the control group (*n* = 9) and naïve mice (*n* = 5) was 109 ± 60, 401 ± 183 and 951 ± 247 mm^3^, respectively (Fig. 4).

### CT26-bearing mice cured by DaRT in combination with CpG, sildenafil and CP resist tumor challenge via tumor-specific immune response

The next question was whether the effect of the above combined treatment was due to a specific immune response against CT26 tumor antigens. Eighteen mice that were cured by DaRT and the three immunomodulators were challenged by an additional dose of the same amount of tumor cells (5 × 10^5^) to their left flank. Ten animals were inoculated with CT26 tumor cells, and eight animals with DA3 tumor cells. Ten naïve mice served as controls. While none of the cured mice inoculated with CT26 cells developed tumors, all cured mice inoculated with DA3 did develop tumors (Table 1, Challenge assay).

**Table 1:** Resistance to challenge of mice cured by DaRT + immunomodulators and specific protection of naïve mice treated with mouse splenocytes

Challenge assay	Mouse source	Challenge cell line	*N*	Number of tumor-bearing mice	Tumor development (%)
	Cured by DaRT + CP + CpG + sildenafil	CT26	10	0	0
	Naïve	CT26	10	10	100
	Cured by DaRT + CP + CpG + sildenafil	DA3	8	8	100
	Naïve	DA3	10	10	100

Winn assay	Splenocytes source	Cell line	*N*	Number of tumor-bearing mice	Tumor development (%)
	Treated by DaRT + CP + CpG + sildenafil	CT26	12	2	17
	Naïve	CT26	12	12	100
	Treated by DaRT + CP + CpG + sildenafil	DA3	9	9	100
	Naïve	DA3	8	8	100

Mice that were cured by DaRT, sildenafil, low-dose CP and CpG (*n* = 18) and naïve mice (*n* = 20) were inoculated with 5 × 10^5^ CT26 or DA3 cells (Challenge assay). Percent of animals that developed tumors following challenge is presented. Naïve mice were injected intradermally with splenocytes from either naïve or CT26-bearing mice treated by DaRT and immunomodulators, coupled with CT26 or DA3 tumor cells in the relation of 45 Ly:1 TC (Winn assay). Percent of tumor development is presented. The presented results are based on cumulative data from two different experiments

In addition, the spleens of mice treated with DaRT + the immunomodulators were harvested 2–5 months after treatment and used to prepare a single-cell suspension. Thereafter, naïve mice were inoculated with DA3 or CT26 tumor cells that were mixed with the harvested splenocytes at 45:1 (splenocytes:tumor cells). Splenocytes of naïve mice served as a control. All mice inoculated with DA3 cells and all mice inoculated with CT26 cells in combination with naïve splenocytes developed tumors, whereas only 17% of the mice inoculated with CT26 cells and the splenocytes taken from mice that were previously treated with DaRT + immunomodulators developed tumors (Table 1, Winn assay). These results demonstrate that the immune response induced by the treatment of DaRT and immonomodulators is mediated by a specific long-term immune memory against CT26 antigens.

## Discussion

The use of alpha radiation to treat solid tumors holds promise as an efficient, precise, and immunostimulating treatment of cancer. Due to the short range of this radiation’s activity and its highly destructive capabilities, the treatment spares the healthy tissue surrounding the tumor on the one hand, and efficiently damages (dividing or non-dividing, normoxic or hypoxic) cells in the malignant tissue on the other. DaRT enables using this type of radiation locally due to its ability to expand the diffusion range of the radiation-emitting atoms, and thus to cover a therapeutically significant volume of the tumor. Importantly, the effect of the treatment is not only local, but also involves activation of a systemic immune response at distant sites [13].

In the present study, we have strengthened our results by employing the tumor-specific challenge and Winn assays. Protection of the mice against tumor cell reinoculation in the opposite lateral flank showed that the antitumor immune response is not only local. Moreover, we showed that DaRT was required for the induction of long-term tumor-specific immune memory and for long-lasting primary tumor rejection when combined with immunomodulation. This suggests a pivotal role for DaRT in the activation and cross-priming of antigen-presenting cells toward the induction of an antigen-specific T-cell response.

Treatment of DA3 breast adenocarcinoma tumors with DaRT in combination with the TLR9 agonist CpG retards local tumor growth, relative to DaRT alone or to CpG alone [13]. Consistent with these results, the current study confirmed that when combined, DaRT and CpG are the main components required for complete tumor rejection in CT26-bearing mice. We also showed that combining DaRT with additional types of TLR agonists similarly improves the antitumor effect. The interaction between TLR agonists and DaRT may be mediated by mechanisms such as cross-presentation of tumor-associated neoantigens from dying cells, which are phagocytosed by dendritic cells [16–19], coupled with cytokine production and danger signals following TLR activation and DNA damage-induced cell death [19–24].

Extending the period of systemic CpG administration did not improve the treatment outcome, implying a limited period of activation or a response plateau. The question of tumor recurrence about 2 weeks after DaRT treatment warrants further study.

The response induced by DaRT and CpG was further enhanced by the inactivation of immune suppressor cells—which impair antitumor activity— such as Tregs [25] and MDSCs [26]. Inhibition of these cells in combination with DaRT and immunoadjuvants resulted in a high percentage of cured animals, suggesting that even when danger signals and dying cells are present in the context of a pathogen, regulatory immune cells still inhibit the immune response. This is probably to protect from false-positive identification of self-antigens presented by cells in the healthy tissue surrounding the pathogen. However, immune adjuvant and inactivation of regulatory immune cells in the absence of DaRT resulted in tumor recurrence, suggesting that tumor ablation by DaRT promotes tumor antigen presentation, and serves to widen the tumor antigen repertoire recognized by the immune system. This is in agreement with previous reports demonstrating the enhancement of MHC1 presentation following radiation of tumor cells [27]. Indeed, this study showed that the cure achieved by the combined treatment was mediated by a specific immune response against tumor-associated antigens. The fact that this setup eliminated immunogenic CT26 tumors implies that it might also be effective with less immunogenic tumors following additional manipulation. For example, using strategies for additional enhancement of the upregulation of tumor-antigen presentation in the treated tumor, prior to the above described treatment.

Radiotherapy acts in synergy with immunostimulation to enhance the distant antitumor immune response [28], and several combinations of radiotherapy and immunotherapy have been tested in humans [28–32]. However, commonly-used types of radiation may cause adverse effects due to their wide range. Therefore, we may conclude that combining DaRT with immunotherapy provides three novel advantages in one treatment: (1) minor radiation-induced adverse effects due to short radiation range; (2) high radiation effectiveness; (3) strong synergy with immunotherapy that enhances the specificity and the distant effects of the antitumor immune response. Since the mortality rate of cancer patients depends mainly on the development of metastases [33], it is highly important to investigate strategies that boost the immune response induced by DaRT. Such strategies may provide a comprehensive treatment for both the local tumor and distant metastases, eventually leading to the possibility of a complete cure of the patients.

DaRT is currently being tested in clinical trials with squamous cell carcinoma patients and has demonstrated high tumor response rates without grade 3 or higher toxicity (NCT03353077). The development of strategies such as those presented here (i.e., tumor ablation by DaRT combined with immunoadjuvants and inhibitors of immunosuppressive cells) is required to move forward in the treatment of this challenging disease.

## Abbreviations

Bq: Becquerel
CP: Cyclophosphamide
DAMP: Damage-associated molecular pattern
DaRT: Diffusing alpha-emitters radiation therapy
Ra: Radium
STR: Short Tandem Repeat
